# Role of BET Inhibitors in Triple Negative Breast Cancers

**DOI:** 10.3390/cancers12040784

**Published:** 2020-03-25

**Authors:** Durga Khandekar, Venkataswarup Tiriveedhi

**Affiliations:** 1 Department of Biological Sciences, Tennessee State University, 3500 John A Merritt Blvd, Nashville, TN 37209, USA; dkhandek@tnstate.edu; 2 Department of Pharmacology, Vanderbilt University, Nashville, TN 37212, USA

**Keywords:** bromodomains, breast cancer, immunotherapy, cancer biology, drug discovery

## Abstract

Bromodomain and extraterminal domain (BET) proteins have evolved as key multifunctional super-regulators that control gene expression. These proteins have been shown to upregulate transcriptional machinery leading to over expression of genes involved in cell proliferation and carcinogenesis. Based on favorable preclinical evidence of BET inhibitors in various cancer models; currently, 26 clinical trials are underway in various stages of study on various hematological and solid organ cancers. Unfortunately, preliminary evidence for these clinical studies does not support the application of BET inhibitors as monotherapy in cancer treatment. Furthermore, the combinatorial efficiency of BET inhibitors with other chemo-and immunotherapeutic agents remain elusive. In this review, we will provide a concise summary of the molecular basis and preliminary clinical outcomes of BET inhibitors in cancer therapy, with special focus on triple negative breast cancer.

## 1. Introduction

**Figure 1:**
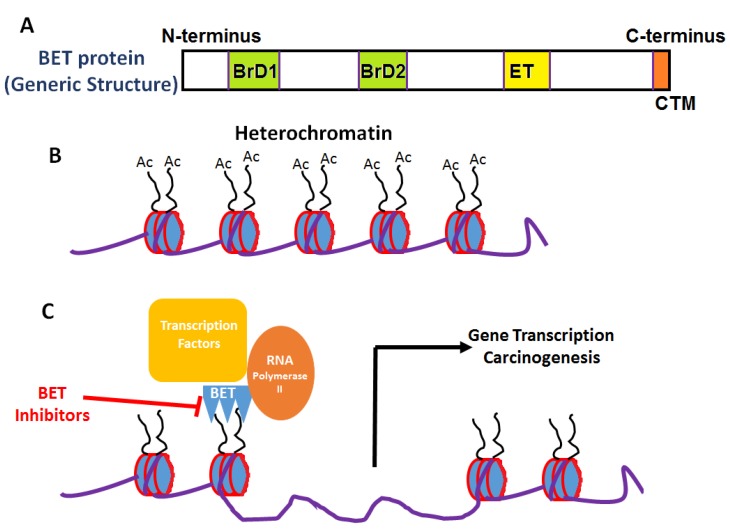
General structure and mechanism of action of BET inhibitors. (**A**) Generic domain structure of the BET protein family. Each BET protein (BRD2, 3, 4 and BRDT) contains two bromodomains (BrD1 and BrD2) and an extra-terminal (ET) domain. An additional carboxy-terminal motif (CTM) is present in BRD4 and BRDT—BET proteins. (**B**) Acetylation of lysine moieties on histones leads to conversion of inactive heterochromatin to active euchromatin. (**C**) BET proteins through their interaction of bromodomain (BRD) motifs with acetylated histones activates transcriptional machinery leading to gene expression and carcinogenesis.

Although all cells in a given organism have the same genomic DNA sequence, characteristic epigenetics allow for the unique identity of individual cell/tissue types to maintain their ability to differentially express genes suitable for their biological function [1]. DNA methylation and covalent histone modifications are the two major hallmarks of epigenetic regulation [2]. Abnormal epigenetic regulatory changes occur in cancers [3]. Under basal conditions, the upstream promoter region on DNA induces only limited gene expression, while higher levels of gene expression observed in cancers require highly regulated promoter–enhancer interactions [4]. Generally, transcription factors are referred to as enhancers, which upon binding with the promoter region induce gene expression by activating transcription machinery consisting of RNA polymerase II. This induction of structural changes in the otherwise cognate promoter region results in cell and microenvironment-specific gene expression [5]. Along with transcription factors, epigenetic mechanisms such as covalent changes to promoter region and histone modifications also exert a key enhancer functionality. As a general rule of thumb, in cancers, there is a wider genomic hypomethylation with localized hypermethylation of tumor suppressor gene promoters [6]. Multiple otherwise normal histone modifications, such as acetylation, methylation, phosphorylation, sumoylation and ubiquitination, along with mutations leading to dysregulation of histones can occur on cancers [7]. The acetylation of functionally active free amino-group of lysine residues in histones by enzymes histone acetyl transferase (HAT) and histone deacetylase (HDAC) induces the formation of transcriptionally active euchromatin [8]. The dysregulation of HAT and HDAC enzymes have been demonstrated in multiple cancers [9]. However, HDAC-based inhibition for cancer therapy has not proved to be very efficient due to the lack of target-specificity. An increasing body of literature evidence suggests that super-enhancer factors [10], in addition to the various mechanisms of action, possess a unique ability to identify acetylated-lysines on histones and modulate the epigenetic enhancer’s function which could span across a long range of genomic DNA to exert stronger transcriptional activation ability through addition of more transcriptional machinery, when compared to regular enhancers (Figure 1). Bromodomain and extraterminal domain (BET) proteins interact with acetylated-lysine portions of histones with a super-enhancer epigenetic role in upregulating gene transcription and thus potentially playing a critical role in carcinogenesis [11]. In this review, we will provide a concise summary of the molecular basis and preliminary clinical outcomes of BET inhibitors in cancer therapy, with special focus on triple negative breast cancer.

## 2. Bromodomain and Extraterminal Domain

The evolutionarily conserved bromodomain (BrD) motifs specifically bind to acetylated lysines in histones [12]. The binding of BrDs to the acetyl group of lysine neutralizes the lysine’s positive charge which then causes the relaxation of heterochromatin in nucleosomes, thereby allowing access to the binding of transcription factors and gene expression [13]. BrDs have a distinctly conserved signature structure with four α-helices, named, Z, A, B and C, with a hydrophobic pocket formed by two flexible linker loop regions (Z/A and B/C) [14]. While the original BrD was identified in Drosophila, in the human proteome, to date, eight sub-families of BrDs with 61 motifs have been identified in 46 proteins [15]. The protein–protein interactions between the hydrophobic pocket of BrDs and acetylated lysines on histones are extremely weak, which makes these motifs enticing targets for development of small molecule drug inhibitors [16].

Bromodomain and extraterminal domain (BET) proteins act as epigenetic super-enhancer modulators with a unique tertiary protein structure generally consisting of two tandem BrDs (BrD1 and BrD2), an extraterminal domain (ET), and a C-terminal domain with the ability to recognize acetylated portions of proteins [11]. The BrD motifs on BET proteins function to facilitate the neutralization of acetylated-lysines and also the recruitment of transcription factors for target gene expression. Literature evidence suggests distinctive functionality for the two BrD motifs of BET proteins, possibly resulting from differential interaction with lysine-acetylated histones or with the transcription factors [17]. For example, in human BRD4, the first BrD motif adheres to the diacetylated H4K5ac/K8ac portion of the histones on the promoter/enhancer region of the target gene, and the second BrD motif enables the recruitment of transcription factors such as pTEFb complex [18]. However, this phenomenon does not seem to be universal for all BET proteins [19]. For BRD3, the first BrD motif is shown to bind with the hematopoietic transcription factor GATA1 [20,21], thereby suggesting differential functionality of the two tandemly arranged BrDs motifs among various BET proteins. Along with BrD motifs, the extra-terminal (ET) domain of BET proteins has shown to play a critical role in overall protein functionality. The ET domain of BRD3 was shown to mediate the identification and eventual interaction of histone and non-histone proteins with BET molecules [22]. 

In addition to acetylated histones, BET proteins have the ability to recognize acetylated transcription factors. The BET family of proteins primarily consists of four proteins, namely, BRD2, BRD3, BRD4, and testis-restricted BRDT [23]. Studies based on in vitro cell culture and plasmid based overexpression of BRD4, have demonstrated that the extraterminal domain of BRD4 is involved in recruiting the positive transcription elongation factor complex (P-TEFb) and initiating the RNA polymerase complex for gene expression [24,25,26]. In addition, a P-TEFb independent gene upregulation by BRD4 has also been reported [27,28]. The accumulating evidence from these diverse data suggest that BRD4 plays a critical role in the transcription initiation and elongation of several genes promoting cell proliferation and cancer progression [18]. BET proteins are also directly involved in the expression of oncogenes such as, c-Myc, which is directly correlated with carcinogenesis [29]. BRD2 was suggested to modulate cell cycle through the expression of cyclin D1 by transcriptional regulatory genes E2F1 and E2F2 [30,31]. Along with BET proteins, BrD motifs have been identified in other proteins such as histone methyltransferase (ASH1L) and the mixed-lineage leukemia (MLL) associated proteins [32]. Along with acetylated-histones, BET proteins are also known to bind with acetylated non-histone proteins such as transcription factors [33]. For instance, BRD4 is shown to bind with the bromodomain region of acetylated-TWIST, a transcription factor associated with embryonic mesodermal development [34,35]. Similarly, BRD4 also modulates the activity of NF-κB complex through its interaction with acetylated RelA [36]. BET proteins can also interact with non-bromodomain motifs of p53, CEBP, etc. [37,38]. Further, although BrD motifs exist in histone acetyl transferases (HATs)—such as p300/CBP-associated factor (PCAF) and cAMP response binding protein (CREBBP)—their exact functionality and molecular regulation of transcription machinery are yet unknown [39,40]. 

## 3. BET Inhibitors

Some of the early BET inhibitors JQ1 and I-BET762 were reported by the Dana-Faber Cancer Institute in collaboration with the Structural Genomics Consortium (SGC) and, GlaxoSmithKline (GSK), respectively [41,42]. JQ1 (thieno-triazolo-1,4-diazepine) was shown to compete with the BRD proteins to bind with the acetylated-lysine residues in various solid organ tumors and hematological malignancies [43,44]. JQ1 causes a significant deletion of interleukin 7 receptor gene (*IL7R*) leading to the downregulation of oncogenes *MYC* and *E2F1* [45,46]. Further, JQ1 has been reported to induce G1-cell cycle arrest and apoptosis in solid tumors [47,48]. 

In tamoxifen-resistant breast cancer, JQ1 through inhibition of WHSC1, a histone H3K36 methyltransferase suppresses ERα signaling pathway causing an anti-tumor effect along with having a synergistic effect with an ER proteolytic fulvestrant [49]. Sengupta et al. reported that JQ1 blocks estrogen (E_2_)-induced transcriptional activation by inhibiting the transition of RNA polymerase II from initiation to elongation phase [50]. Similarly, a combination of JQ1 with mocetinostat, a HDAC inhibitor, caused the inhibition of the RAS/MAPkinase signaling pathway, leading to decreased cell proliferation in both ER+ and triple negative breast cancers (TNBC) [51]. Selectively in TNBC, drug treatment with JQ1 exerted the downregulation of the cell cycle transcription factors Forkhead box M1 (*FOXM1*) and Lim domain only 4 (*LMO4*), angiogenic factors vascular endothelial growth factor A (*VEGF-A*) and carbonic anhydrase 9 (*CA9*), thus reducing cell proliferation, angiogenesis and metastasis [52]. 

A BRD2/3/4 inhibitor, OTX015 (MK-8628) is currently under clinical trial for evaluation in dose-finding studies and safety in TNBC [53]. In vitro cell culture studies with OTX015 caused inhibition of cellular proliferation and cell cycle arrest in leukemia cell lines [54]. I-BET151 (GSK) was reported to inhibit constitutively active JAK2 in glioblastoma leading to G1 arrest of cell cycle [55]. PFI-1 is a BET inhibitor with dual inhibition of BRD2 and BRD4 has been reported to exert anti-proliferative effect on leukemic cell lines [56]. Treatment with PFI-1 induced inhibition of *MYC* expression through the downregulation of oncogene *Aurora kinase B*, leading to cancer cell apoptosis [56]. The BRD4 inhibitor, MS436, was shown to exert anti-inflammatory effect on macrophages through downregulation of NF-κB mediated IL-6 expression [57]. However, there are no reports on the role of MS346 in cancer. Along with these, several newly discovered BET/BrD inhibitors, such as FT-1101, CPI-0610, BAY 1238097, INCB054329 TEN-010, BAY-299, etc., are currently under various phases of cancer clinical trials [58].

Numerous studies have reported that broad chemical inhibition of both BrD motifs of BET molecules effectively block genome-wide transcription of multiple key cancer and immune regulatory genes. However, the use of selective inhibitors of single BrDs could have a distinctive functional advantage. Gacias et al., have reported that specific BrD motif inhibition using a selective chemical inhibitor such as olinone to preferentially block one of the two BrDs on BET proteins inhibits lineage differentiation in neural oligodendrocytes [59]. A similar strategy targeting a single BrD motif on BET proteins could be of great interest for its future anti-cancer impact. The distinctive and unique ligand-binding selectivity between the two BrD motifs has been attributed to a few amino acid residues that distinguish the first and second BrDs within each BET protein, while all of them share nearly similar residues at the acetylated-lysine binding pocket [59]. More efforts are needed in this direction to enhance the current understanding of the specific molecular functions of the individual BrD motifs of BET proteins to develop more specific drug-targets.

**Figure 2:**
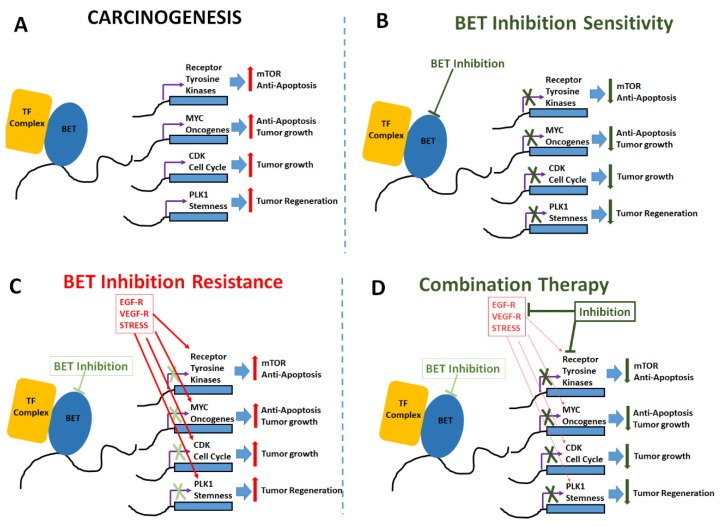
Mechanisms of BET inhibitor activity, resistance and combinatorial application. (**A**) Carcinogenic changes mediated by upregulation of tumorigenic transcription factors, anti-apoptotic genes, oncogenes and cell cycle inducers following epigenetic enhancement by BET proteins. (**B**) BET inhibitors induce anti-tumor effect by enhancing apoptosis and reducing cell proliferation. (**C**) Cell adaptation mechanisms to overcome BET inhibition by upregulation of receptors for epidermal growth factor (EGF-R), vascular endothelial growth factor (VEGF-R) and other stress mediated factors such as HIF1α etc. (**D**) Combinatorial treatment with addition of drugs targeted at mTOR pathway and other oncogenic pathways along with BET inhibition to enhance anti-cancer impact.

Several mechanisms which enhance the chemotherapeutic sensitivity following BET inhibitor application have been noticed (Figure 2). The mammalian target of the rapamycin (mTOR) pathway, PI3K/AKT/mTOR, has been shown to be an important chemotherapeutic drug target in TNBC. Everolimus, a selective inhibitor of mTOR pathway, was suggested to exert anti-tumor efficient against the basal-like subtype of TNBC cell lines. However, in clinical breast cancer studies, the use of this mTOR inhibitor as a single-agent has resulted in minimally efficiency [60]. Studies by Stuhlmiller et al. have demonstrated that JQ1 enhanced the everolimus sensitivity of TNBC cells, leading to reduced cell proliferation and enhanced apoptosis [61]. Similarly, studies by Vazquez et al. have demonstrated that a combination of OTX015 with everolimus has enhanced the drug sensitivity in TNBC pre-clinical models [53]. The overexpression of the *MYC* oncogene is frequently reported in TNBC [62]. Efforts to directly target MYC expression and functionality have not been successful. Furthermore, there is evidence to suggest that BRD4-induced up-regulation of c-MYC played a critical role in inducing resistance to everolimus in ER+ breast cancer cells [63]. BET inhibitors OTX015 and JQ1 have been reported to induce *c-MYC* down-regulation in several cancer types including TNBC [64,65].

There is increasing evidence that tumor-initiating stem cells (TISCs) play a critical role in tumor recurrence and treatment failure in several solid and hematological cancers [66]. Treatment failure to paclitaxel and cisplatin in TNBC patients is suggested to be associated with TISC generation [67,68]. BET proteins have been demonstrated to play a crucial role in the stem-functionality of TISCs. Studies by Vazquez et al. have demonstrated that OTX015 down-regulated the expression of stem cell functionality associated genes *NANOG* and *OCT4*. Similarly, Horne et al. have demonstrated the downregulation of NANOG expression following treatment with JQ1 on murine embryonic stem cells [67]. The binding of BRD4 with the promoter region of stemness associated gene *WNT5A* is shown to enhance the tumor cell regeneration and invasiveness of TISCs in in basal-like breast cancer [34]. The BET inhibitor JQ1 is also known to inhibit the stem cell associated with acute myeloid leukemia [69]. All these data strongly suggest a potential role of BET inhibitors in targeting TISCs in various cancers. BET inhibitors were also suggested to inhibit stem cell functionality through inhibition of JAK/STAT pathway [70,71]. In TISCs, the polo-like kinase (PLK1) induces the M-phase of the cell cycle [72,73]. Studies by Mao et al., have demonstrated that the BET inhibitor produced a cell cycle arrest at G1, while the volasertib, a PLK1 inhibitor, induced cells to arrest at M-phase [74,75]. Further BET inhibitors induced not only arrest at G1-cell cycle, but also reduced the levels of kinases such as PLK1 involved in mitosis [53]. Taken together, these data suggest that BET inhibition is an attractive targeted therapy against TISCs in TNBC patients.

Currently, there are very limited BET inhibitor based clinical trials in TNBC. Of the 25 clinical trials (listed in *www.clinicaltrials.gov*), only four trials included breast cancer (Table 1), while the remaining 21 trials (not provided in the table) were on hematological malignancies. Unfortunately, the preliminary results from these studies are not promising. The majority of these preclinical studies indicate resistance to BET inhibitors. The cancer resistance does not seem to be due to changes in the BET protein’s gene expression pattern such as copy-number changes or somatic mutations on gate-keeper genes induced by specific BET inhibitors [76]. For example, in TNBC preclinical proteomic studies, BET inhibitor resistance was suggested to be due to the downregulation of protein phosphates 2A (PP2A) leading to hyper-phosphorylation of BRD4 and the enhanced interaction and activity of MED1, a mediator of RNA polymerase II, leading to the upregulation of transcription machinery and cell proliferation [76,77]. Similarly, in other hematological cancers, BET inhibitor treatment was shown to induce delayed WNT/β-catenin signaling-mediated MYC oncogene expression [78,79]. Furthermore, pre-existing mutations in PIK3CA in breast cancers is associated with BET inhibitor resistance [80,81,82]. Furthermore, pre-existing LKB1 and KRAS mutations in lung cancer have been associated with BET inhibitor resistance [83,84].

Structure activity relationship studies targeted at the better optimization of BET inhibitors was met with some major road-blocks, predominantly arising from the lack of current understanding of bromodomain motifs [85]. For example, unlike pan-bromodomain inhibitors, selective bromodomain-1 (MS-436, Olinone, and BI-2536) and bromodoamain-2 specific (RVX-208 and RVX-297) compounds were developed [86,87,88,89]. However, these individual sub-motif specific compounds have led to unexpected outcomes. Olinone, a bromodomain-1 specific BET inhibitor caused terminal primary differentiation of oligodendrocytes, while pan-BET inhibitors induced inhibition of oligodendrocyte growth and activity [59]. Further, BET inhibition was noted as an important off-target effect. Drugs such as dinaciclib (CDK inhibitor), TG101209 (JAK2 inhibitor) and BI-2536 (PLK inhibitor) have shown a strong BET inhibitor potential [90,91,92]. These off-target effects pose an opportunity to use these compounds for their dual-effect versus a challenge to limit their use due to unintended side effects. More stringent dose-dependent clinical studies should be performed to evaluate the utility of this off-target effect.

## 4. Challenges

While original functional and genomic studies on BRD4 were performed on NUT midline carcinoma, because of the lower incidence of this disease (as compared to other cancers), diverse scientific opinions exist on a more generalized applicability in other malignancies [94]. In hematological malignancies, based on shRNA-based knock-down and other proteomic data, BRD4 is correlated with the rearrangement of the mixed lineage leukemia (*MLL1*) gene (renamed as lysine specific methyl transferase 2A, *KMT2A*) [95]. Based on these studies, several clinical trials were initiated to demonstrate the efficacy of BET inhibitors in hematological malignancies. In spite of the initiation of BET inhibitor-based clinical studies, the precise genetic signatures and gene pathway clusters modulated by BET inhibitors have been areas of debate. Studies with JQ1/iBET on various cell lines from solid organ tumors demonstrated divergent results with the induction of terminal cell differentiation in some cancer cell lines to apoptosis in other cell lines [96,97]. Large scale profiling studies demonstrated that BET inhibitors induced the suppression of some oncogenes such as *MYC*, *BCL2*, *CDK6*, etc., while they had no impact on house-keeping genes [15,98].

In preclinical animal models of cancer, BET inhibitors have shown some unique effects on normal tissues too. Nicodeme et al. have demonstrated that treatment of animals with iBET helped overcome septic shock due to their anti-inflammatory effect and ability to inhibit the expression of inflammatory cytokines leading to immunosuppression [41]. In cardiac studies, BET inhibitors were able to inhibit cardiomyocyte damage and overcome pressure-overload effect in hypertension and congestive heart failure models [99]. In addition to these effects, BET inhibitors have also been shown to induce temporary infertility in men with their ability to inhibit spermatogenesis in the testes [100,101]. In addition, BET inhibitors are correlated with memory impairment, autism-like disorder and worsened bacterial co-infections in HIV-mediated immunosuppressive disorders [102,103]. In clinical studies, several important side-effects have been noted in patients being treated with OTX015, TEN-010, and CPI-0610 based BET inhibitor therapy [17,104]. Patients treated with high-dose OTX015 (120–160 mg/day) had adverse side-effects, such as thrombocytopenia, gastrointestinal bleeding and severe fatigue (Table 2).

## 5. Future Role of Novel Drug Design and Combinatorial Therapy

To-date, application of BET inhibitors has resulted in limited success. The only clinical trials with OTX015 (MK-8628, NCT02259114) in TNBC were discontinued due to a lack of clinical efficacy, in spite of its efficiency in preclinical TNBC models [53]. Therefore, more research is needed for the discovery of drugs which would exert more a sustained and efficient inhibition of BET proteins. Compounds with bivalent efficiency to simultaneously block two bromodomain motifs such as AZD5153, biBET and MT1 have demonstrated favorable outcomes in preclinical cell culture-based studies [105,106]. The development of dual-action proteolysis targeting chimera-based compounds with a BET inhibitor compound merged with protein degrading E3 ubiquitin ligase proteasome complex allows for selective degradation of BET proteins [107,108]. Along these lines, the original BET inhibitor BETi-211 was modified to BETd-246 and BETd-260 to include E3 ubiquitin ligase activity and has shown more efficient outcomes in preclinical TNBC studies [109]. Similarly, dual-functional compounds such as ARV-825 and ARV-771 have shown significantly higher anti-cancer effects over BET inhibitors such as, JQ1 or OTX015 [110].

Preclinical studies in various cancer models have demonstrated an apparent combinatorial synergism of BET inhibitors with various previously anti-cancer chemo-and immunotherapeutic agents (Table 3). In breast cancer studies, a combination of BET inhibitors with PI3K inhibitors demonstrated significantly decreased expression of downstream PI3K signaling genes such as EGFR and IGF growth factors, leading to reduced cell proliferation, as compared to treatment with PI3K inhibitor alone [53,61,82]. Similarly, a combination of BET inhibitor with PARP inhibitor, olaparib, has demonstrated significant reduction in transcription of BRAC1 and RAD51 genes [111,112]. In hematological cancers, active clinical trials are underway to study the combinatorial benefit of combining BET inhibitors with BCL2 inhibitors [113]. Interestingly, some of the known anti-cancer kinases inhibitors which target PLK1 and JAK2 (TG-101348) have also shown BET inhibitor capability [114]. Further dose-dependent clinical studies are needed to better understand the multi-functional efficiency of these drugs. Studies by Hogg et al. have demonstrated that treatment with BET inhibitors decreased the expression of PD-L1 in tumor cells through the inhibition of BRD4 binding to *CD274* locus on chromosome 9 [115]. These data suggest a potential role of a combinatorial therapy of immune-modulating agents such as anti-PD1, anti-CTLA4 and CAR-T cells with BET inhibitors [116]. In the context of preclinical breast and prostate cancer models, BET inhibitors have suggested a combinatorial benefit with hormone receptor-modulating agents fulvestrant and enzalutamide [117]. All these various combinations should undergo stringent clinical trials to prove clinical applicability.

## 6. Conclusions

In conclusion, despite promising evidence from preclinical models, the clinical application of BET inhibitors remains elusive. Similar to chemotherapeutic agents such as alkylating and cell-cycle disrupting agents, BET inhibitors target transcriptional machinery with a higher impact of rapidly dividing cancer cells over normal cells. Although the final results from several of the BET inhibitor-based clinical trials are eagerly awaited, there could be detrimental side-effects, possibly explaining their limited success in the initial evidence from clinical trials. However, in spite of these challenges, we think that BET inhibitors have a promising role in combinatorial therapy and the future development of novel dual-BRD-motifs targeting inhibitors. Further studies are needed to determine the specific biomarkers which would implicate the long-term success of BET inhibitors in the p therapeutic application of a cancer patient, paving the way for personalized medicine.

## Figures and Tables

**Table 1:** Clinical trials with BET inhibitors on triple negative breast cancers. (# Identifier number on www.clinicaltrials.gov).

Drug	Identifier #	Tumor Type	Clinical Phase	Status
MK-8628/OTX105	NCT02259114	NUT Midline Carcinoma	Phase IB	Completed [93]
		Non-small Cell Lung Cancer		
		Castrate-resistant Prostate Cancer		
		Pancreatic Ductal Adenocarcinoma		
GSK525762	NCT01587703	All solid tumors, Midline	Phase 1	Active, not recruiting
MK-8628	NCT02698176	NUT Midline Carcinoma	Phase 1	Terminated
		Non-small Cell Lung Cancer		
		Castrate-resistant Prostate Cancer		
		Pancreatic Ductal Adenocarcinoma		
INCB054329	NCT02431260	Solid Tumors and Hematologic Malignancy	Phase 1 Phase 2	Terminated

**Table 2:** Toxicities reported from clinical trials with BET inhibitors.

Therapeutic Agents	Malignancies	Toxicities
MK-8268/OTX-015 [88]	Relapsed/refractory leukemia	diarrhea, fatigue, anorexia. Toxicities also included dysgeusia, abdominal pain, decreased clotting factor VII
MK-8628/OTX015 [89]	Relapsed/refractory lymphoma or multiple myeloma	thrombocytopenia, neutropenia, hyponatremia; diarrhea, dysgeusia, fatigue, anemia
MK-8628/OTX-015 [90]	NUT midline carcinoma	thrombocytopenia, nausea, dysgeusia, hyperglycemia, fatigue
BAY1238097 [91]	Advanced solid tumors or NHL	headache, vomiting, low back pain, Recurrent headaches

**Table 3:** Various combinations used with BET inhibitors on pre-clinical models.

Combination Therapy	Pre-Clinical Models Tested
JQ1 and FLT3-TK1 [118]	Immunodeficient mice injected with OCIAML3 or MOLM13 cells
JQ1/dBET1 and Ponatinib [119]	Colon (HCT116, HT29), breast (MCF-7, SKBR3) and ovarian (A2780, SKOV3) cancer cells
I-BET151 and panobinostat [120]	Immunodeficient mice injected with patient-derived melanoma cells resistant to vemurafenib
JQ1 and panobinostat [121]	Syngeneic orthotopic murine tumors, SK-N-BE (2) neuroblastoma cells
JQ1 and romidepsin [48]	Murine tumor models of NT2/D1 and NCCIT embryonal carcinoma
JQ1 and rapamycin [122]	Immunodeficient mice injected with MNNG/HOS osteosarcoma cells
CPI203 and rapamycin [123]	Immunodeficient mice injected with BON-1 pancreatic neuroendocrine tumor cells
JQ1 and trametinib [124]	Immunodeficient mice injected with ES2 ovarian clear cell carcinoma cells
JQ1 and vemurafenib [125]	Immunodeficient mice injected with A375 melanoma cells
JQ1 and fulvestrant [49]	Immunodeficient mice injected with tamoxifen-resistant MCF7 breast cancer cells
I-BET151 and lapatinib [126]	Immunodeficient mice injected with Her2þ BT474 breast cancer cells
JQ1 and lenalidomide [127]	Immunodeficient mice injected with BC-3 lymphoma cells
JQ1 and unidentified PD-1 inhibitor [128]	KRASmt NSCLC murine tumor model
RVX2135 and ATR inhibitor AZ20 [129]	Syngeneic λ820 and λ2749 murine Myc-induced lymphoma xenografts
